# Evaluation of lipid quantification accuracy using HILIC and RPLC MS on the example of NIST® SRM® 1950 metabolites in human plasma

**DOI:** 10.1007/s00216-020-02576-x

**Published:** 2020-04-02

**Authors:** Mike Lange, Maria Fedorova

**Affiliations:** 1 Institute of Bioanalytical Chemistry, Faculty of Chemistry and Mineralogy, University of Leipzig, Deutscher Platz 5, 04103 Leipzig, Germany; 2 Center for Biotechnology and Biomedicine, University of Leipzig, Deutscher Platz 5, 04103 Leipzig, Germany

**Keywords:** Lipidomics, Human blood plasma, UHPLC, HILIC, RPLC, Quantification, Mass spectrometry

## Abstract

Lipidomics analysis for large-scale studies aiming at the identification and quantification of natural lipidomes is often performed using LC–MS-based data acquisition. However, the choice of suitable LC–MS method for accurate lipid quantification remains a matter of debate. Here, we performed the systematic comparison between two HRAM-MS-based quantification workflows based on HILIC and RPLC MS by quantifying 191 lipids from five lipid classes in human blood plasma using deuterated standards in the “one ISTD-per-lipid class” approach. Lipid quantification was performed considering all necessary isotopic corrections, and obtained correction factors are illustrated. Concentrations of lipids in NIST® SRM® 1950 human blood plasma determined by the two methods were comparable for most of the studied lipid species except for highly unsaturated phosphatidylcholines (PC). A comparison of lipid concentrations to consensus values determined in a previously published multi-laboratory study illustrated possible “overestimation” of concentrations for these highly unsaturated lipids by HILIC MS. We evaluated the influence of lipid loading amounts as well as the difference between quantified lipid and internal standard concentrations on the HILIC MS quantification results. We conclude that both HILIC and RPLC HRAM-MS workflows can be equally used for accurate lysophosphatidylcholine (LPC), lysophosphatidylethanolamine (LPE), phosphatidylcholine (PC), phosphatidylethanolamine (PE), and sphingomyelin (SM) lipid quantification, despite significant differences in the concentration of highly unsaturated PC lipids which need to be addressed by establishing response factors to account for the differences in degree of lipid unsaturation.

**Electronic supplementary material:**

The online version of this article (10.1007/s00216-020-02576-x) contains supplementary material, which is available to authorized users.

**Figure:**
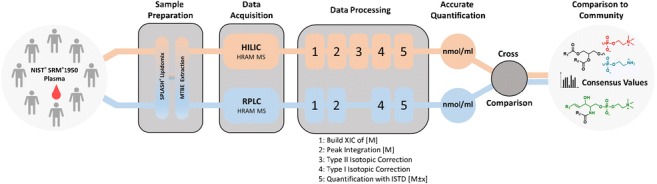
Graphical

## Introduction

Lipids play crucial roles in a plethora of physiological functions ranging from energy storage, cellular compartmentalization, regulation of protein function to signaling [1]. In order to unravel lipid function, it is of utmost importance to identify and quantify single lipid molecular species in complex biological mixtures. Lipidomics studies based on the application of liquid chromatography (LC) coupled online to mass spectrometry (MS) are often used to identify and quantify lipids in a complex biological sample. The lipidomics community nowadays underlines the importance of reporting quantitative values for lipid species as the only way to facilitate inter-laboratory and inter-study comparison of results at least for most often studied lipidomes such as human blood plasma [2].

The clear need for protocol standardization to ensure quality of reported lipid concentrations is significantly challenged by the fact that there are more than one MS-based method used for lipid analysis. Several quantification approaches have been developed implementing MS without (shotgun) or with prior chromatographic separation (LC–MS) in which MS quantification is based on adding compound-specific internal standards (ISTD) [3–5]. Quantitative mass spectrometry data may be acquired in a targeted or untargeted fashion whereas each acquisition method has certain advantages and disadvantages. Targeted methods (e.g., *single ion monitoring (SIM), multiple/parallel reaction monitoring (MRM/PRM)*) need prior knowledge of the sample matrix and specific LC and MS properties of the analytes accompanied by a limited number of quantification targets [6–8]. Untargeted data acquisition modes (e.g., *full-MS, data-dependent (DDA-MS), and data-independent acquisition MS (DIA-MS)*) [9–11] allow for the quantification of theoretically all analytes present in a sample and do not require prior knowledge of the sample matrix; thus, they are well suited for *Discovery Lipidomics* approaches. Furthermore, feature quantification may be performed on intact precursor ions enabling excellent chromatographic peak integration and sensitivity at the cost of decreased specificity (*Full-MS, SIM*) or on analyte-specific fragment ions increasing specificity (*SWATH-MS, PRM/MRM*) but complicating quantification due to a limited number of data points over chromatographic peak.

Accuracy of quantification also relies on the close similarity between physicochemical properties of added ISTD and the native lipid that is ought to be quantified. For that purpose, a chemically identical, isotopically labeled ISTD can be added to allow for the *absolute quantification* of a single lipid molecular species. It is experimentally evident that up to several thousand lipids can be present in a biological sample, and it is therefore not possible so far to obtain an ISTD for each single molecular lipid species. Usually, non-native lipid class-specific ISTDs (isotopically labeled, odd-numbered, or the one absent in the sample) [12] are added in a similar concentration as the lipids to be quantified which allow for *accurate quantification* in the so-called one ISTD-per-lipid class approach. However, it should be considered that native lipids and added ISTD are structurally not identical, and thus, several correction algorithms have to be implemented during the data processing workflow to make up for those structural differences [13–15]. Additionally, other factors are influencing the analytical response in MS experiments and therefore the analytical accuracy: (i) chromatographic matrix effects originating from different elution times; (ii) molecular species-dependent ionizability, surface activity, and adducts formation during the ESI process; and (iii) molecular species-dependent in-source fragmentation [13].

In LC–MS-based lipidomics, the two most widely applied separation modes are reversed-phase liquid chromatography (RPLC) and hydrophilic interaction chromatography (HILIC) [16] which have fundamentally different separation modes. RPLC is separating lipids mainly based on their fatty acyl/alkyl chain hydrophobicity whereas HILIC is separating lipids based on their headgroup polarity leading to coelution of all lipids of a specific lipid class [17, 18]. MS response of a lipid is mainly determined by its lipid class, the fatty acid chain length, and unsaturation as well as the solvent in which it is ionized [19, 20]. Therefore, HILIC-based MS quantification is capable of diminishing elution-dependent matrix effects due to coelution of the ISTD with the lipids of the same class. On the other hand, due to the coelution of all species within a defined lipid class, possible ionization suppression can occur favoring detection of high abundant lipid species over the low abundant ones. RPLC-based MS methods allow for quantification of isomeric lipids of the same class. However, since RPLC-separated lipids and the corresponding ISTD are distributed over a broad retention time range with quite different solvent compositions, increased matrix effects might decrease the accuracy of quantification results. Surprisingly, yet there has been no direct comparison of HILIC- and RPLC-based MS methods for their accuracy to quantifying lipids. To close this gap, we compare quantitative values for 191 lipids from five different lipid classes (LPC, LPE, PC, PE, and SM) in NIST® SRM® 1950 Metabolites in Frozen Plasma determined by RPLC and HILIC MS quantitative workflows.

## Materials and methods

### Chemicals

Acetonitrile (MeCN), 2-propanol (*i*-PrOH), methanol (MeOH), and formic acid (all ULC/MS-CC/SFC grade) were purchased from Biosolve (Valkenswaard, Netherlands). Chloroform Emsure®, *tert*-butyl methyl ether (MTBE) (≥ 99%), butylated hydroxytoluene (≥ 99%), and the NIST® SRM® 1950 Metabolites in Frozen Plasma were purchased from Sigma-Aldrich (Taufkirchen, Germany). Ammonium formate (NH_4_HCO_2_) and ammonium acetate (NH_4_OAc) (both MS grade) were purchased from Fluka Analytical (München, Germany). SPLASH® LIPIDOMIX® was purchased from Avanti Polar Lipids Inc. (Alabaster, AL, USA). Water was ultrapurified by an ELGA PURELAB Ultra Analytic (Berlin, Germany) instrument delivering water quality ≥ 18.2 MΩ-cm.

### Preanalytics

NIST plasma was delivered frozen in 1-mL tubes and stored at − 80 °C until further processing. For lipid extraction, NIST plasma was thawed on ice for 1 h with subsequent vortexing. Plasma was aliquoted in 50-μL aliquots and stored at − 80 °C.

### Lipid extraction

Five aliquots of NIST plasma were thawed by incubating tubes containing 50 μL plasma on ice for 1 h. SPLASH® LIPIDOMIX® was added (ratio 1:10, SPLASH:plasma, v/v) and incubated on ice for 15 min. Lipids were extracted as described before [14] by adding ice-cold MeOH (375 μL) and MTBE (1250 μL) with subsequent vortexing. Homogenates were incubated for 1 h at 4 °C (orbital shaker, 32 rpm). Phase separation was induced by addition of H_2_O (375 μL), vortexed and incubated for 10 min at 4 °C (orbital shaker, 32 rpm). Afterwards, the sample was centrifuged to separate the organic and aqueous phases (10 min, 4 °C, 1000×*g*). The organic phase was collected into a new tube. Re-extraction of the remaining aqueous phase was performed by addition of MTBE/MeOH/H_2_O (4/1.2/1, v/v; 500 μL) with subsequent vortexing. The samples were centrifuged (10 min, 4 °C, 1000×*g*), organic phases were combined, and the solvent was removed in vacuo (Eppendorf concentrator 5301, 1 ppm). A quality control (QC) sample was prepared by mixing obtained lipid extracts in an equivolumetric manner and was used for method development and monitoring of analytical accuracy.

### Liquid chromatography

Subsequently, the lipid extract was reconstituted in pure *i*-PrOH (200 μL) by vigorous vortexing. Lipids were diluted with *i*-PrOH to a final concentration of 0.03 μL_plasma_/μL_i-PrOH_ of which 5 μL was injected onto the column corresponding to 0.15 μL_plasma_.

Reversed-phase liquid chromatography (RPLC) was carried out on a Vanquish focused^+^ (Thermo Fisher Scientific, Bremen, Germany) equipped with an Accucore C18 column (150 × 2.1 mm; 2.6 μm, 150 Å; Thermo Fisher Scientific, Bremen, Germany). Lipids were separated by gradient elution with solvent A (MeCN/H_2_O, 1:1, v/v) and B (*i*-PrOH/MeCN/H_2_O, 85:10:5, v/v), both containing 5 mM NH_4_HCO_2_ and 0.1% (v/v) formic acid. Separation was performed at 50 °C with a flow rate of 0.3 mL/min using the following gradient: 0–20 min—10 to 86% B (curve 4), 20–22 min—86 to 95% B (curve 5), 22–26 min—95% isocratic, and 26–26.1 min—95 to 10% B (curve 5) followed by 5 min re-equilibration at 10% B.

Hydrophilic interaction chromatography (HILIC) was carried out on a Vanquish focused^+^ (Thermo Fisher Scientific) equipped with an Acquity UPLC BEH HILIC Si column (100 × 1.0 mm, 1.7 μm, 130 Å; Waters Corp.). Lipids were separated as described previously [3] by gradient elution with solvent A (MeCN/H_2_O, 96:4, v/v) and B (H_2_O) both containing 7 mM NH_4_OAc. Separation was performed at 40 °C with a flow rate of 0.15 mL/min using the following gradient: 0–10 min—0 to 10% B (curve 5) and 10–10.1 min—10 to 0% B (curve 5) followed by 5 min re-equilibration at 0% B.

### Mass spectrometry

Both UHPLC experiments were performed using a Q Exactive Plus Hybrid Quadrupole-Orbitrap mass spectrometer (Thermo Fisher Scientific, Bremen, Germany) equipped with a HESI probe. Mass spectra were acquired in the mass range of *m*/*z* 100–1500 in positive and negative modes with the following ESI parameters: sheath gas—40 L/min, auxiliary gas—10 L/min, sweep gas—1 L/min, spray voltage—2.5 kV, spray current—10 μA, capillary temperature—300 °C, S-lens RF level—35, and aux gas heater temperature—370 °C.

For lipid identification, parallel reaction monitoring (PRM) using *m*/*z* of 191 previously reported blood plasma lipids (LPC, PC, LPE, PE, and SM) as precursors was used in negative mode for phospholipids or positive mode for SM at the resolution of 17,500 at *m*/*z* 200, AGC target of 2e5, and a maximum injection time of 40 ms. The isolation window for precursor selection was 1.2 *m*/*z*, and normalized stepped collision energy (10, 20, and 30 eV) was used for HCD. Data were acquired in profile mode.

For quantification, data was acquired in full MS mode only in positive polarity at the resolution of 140,000 at *m*/*z* 200, AGC target of 1e6, and maximum injection time of 100 ms in the mass range from *m*/*z* 100 to 1500. Data were acquired in profile mode.

### Data processing

Lipids were identified based on MS^2^ fragmentation patterns. A lipid identification was accepted when the mass difference between expected and measured *m*/*z* was below 5 ppm and characteristic fragments for specific lipids could be detected.

For quantification, raw data sets of full MS measurements were processed using TraceFinder™ 4.1 (Thermo Fisher Scientific, Bremen, Germany). For all investigated lipid classes, only the monoisotopic mass peaks of protonated lipid adducts *[M + H]*^*+*^ were detectable and area under the curve (AUC; also referred to as peak integral or an extracted ion chromatogram) was determined using the following settings: mass tolerance—5 ppm, area noise factor—5, peak noise factor—10, baseline window—150, and *S*/*N* ≥ 3 using ICIS detection algorithm.

A detailed description of the quantitative workflow is presented in Fig. S1 in the Electronic Supplementary Material (ESM). Type I isotopic correction for ^13^C-abundance was performed with an excel macro as described previously [3] using the following correlation [15]:

$$ {\mathrm{AUC}}_{n(k)\ \mathrm{total}}={\mathrm{AUC}}_{n(k)}\left(\ 1+0.0109n+\frac{0.0109^2n\left(n-1\right)}{2}\right), $$

AUC_*n*(*k*) total_ = total ion area under curve, AUC_*n*(*k*)_ = quantified area under curve of monoisotopic mass, *n* = no. of C atoms, *k* = no. of double bonds

Type II isotopic correction for overlapping, coeluting isotopologues was performed as previously described using the following equation [15]:

$$ {\mathrm{AUC}}_{n(k)\ \mathrm{total}}={\mathrm{AUC}}_{n(k)}-{\mathrm{AUC}}_{n\left(k+1\right)}\left(\frac{0.0109^2n\left(n-1\right)}{2}\right) $$

AUC_*n*(*k*) total_ = total ion area under curve, AUC_*n*(*k*)_ = quantified area under curve of monoisotopic mass, *n* = no. of C atoms, *k* = no. of double bonds

Accurate quantification of lipid species was performed by relating the corrected AUC values of the lipid species to the corrected AUC of lipid class ISTD.

$$ {C}_{\mathrm{lipid}}=\frac{{\mathrm{AUC}}_{\mathrm{lipid}}}{{\mathrm{AUC}}_{\mathrm{ISTD}}}\ast {C}_{\mathrm{ISTD}} $$

*C*_lipid_ = concentration of lipid species, *C*_ISTD_ = concentration of ISTD, AUC_lipid_ = corrected area under curve for lipid species, AUC_ISTD_ = area under curve for ISTD

The obtained lipid concentrations were compared to the established consensus values using LipidQC [21] software as previously described.

Quantitative reliability of obtained lipid concentrations was assessed by determination of the relative standard deviation (RSD) in between replicates. A lipid concentration was chosen to be reliably quantified if RSD was ≤ 20% in accordance with the definition of the upper and lower limits of quantification provided by the FDA [22].

## Results and discussion

### Evaluation of quantitative lipidomic workflow

The aim of this work was to compare HILIC and RPLC-MS workflows in their ability to quantify lipids from biological matrices and to assess systematic differences between those methods arising during the quantitation. Therefore, the NIST® SRM® 1950 Metabolites in Frozen Plasma was chosen as the reference matrix since consensus values for 339 different lipids were already established and validated by the community [2]. The SPLASH® LIPIDOMIX® was used as a ready, commercially available mixture of lipid class-specific internal standards (ISTD) as it was designed to match lipid concentrations in human plasma. Lipid extracts were separated by either HILIC or RPLC and measured under identical high-resolution accurate-mass (HRAM) mass spectrometry settings with a standardized data processing workflow.

Here, we focused on quantification of five lipid classes including lysophosphatidylcholines (LPC), lysophosphatidylethanolamines (LPE), phosphatidylcholines (PC), phosphatidylethanolamines (PE), and sphingomyelins (SM) as the most abundant lipids for which consensus values have been established and that show good chromatographic retention by both, RPLC and HILIC MS methods, used in our study (ESM Table S1) [23, 24]. Lipid identities were confirmed in the PRM experiment, and corresponding retention times were determined for lipids with reported consensus values [2]. Unambiguous identification was based on accurate mass and the characteristic fragmentation behavior upon HCD as reported previously [25]. Subsequently, lipid extracts were separated by both methods using *full-MS* scans at the resolution of 140,000 (at *m*/*z* 200) to ensure MS separation of isobaric lipid species. Quantification workflow was subdivided into several steps:

#### (1). Peak area integration of extracted ion chromatograms of the monoisotopic precursor

The quantitative data processing workflow starts with generating extracted ion chromatograms (XIC) for the monoisotopic mass (*[M]*) of identified lipids with a mass accuracy of 5 ppm within a determined retention time window. Monoisotopic XIC area integration can be routinely performed by different software tools, and here, TraceFinder v4.1 was used. Lipid concentrations could be potentially determined by relating the peak area of the native lipid to the peak area of the corresponding class-specific ISTD. However, several important correction factors have to be implemented to increase the accuracy of lipid quantification as illustrated below (ESM Table S2).

#### (2). Type I isotopic correction

The intensity of *[M]* relative to the total ion intensity (summed intensity of all isotopologues *[M]*, *[M + 1]*, *[M + 2]*) differs depending on the number of carbon atoms in the molecule due to isotopic effects caused by the natural ^13^C abundance. Since ISTD and native lipids are not identical on the molecular level (different number of carbon atoms), using area under the curve (AUC) for only *[M]* will impact accuracy of the quantification as it does not reflect the total ion abundance. Consequently, the total ion abundance of both the ISTD and the native lipid has to be taken into account to ensure accurate quantification.

**Fig. 1:**
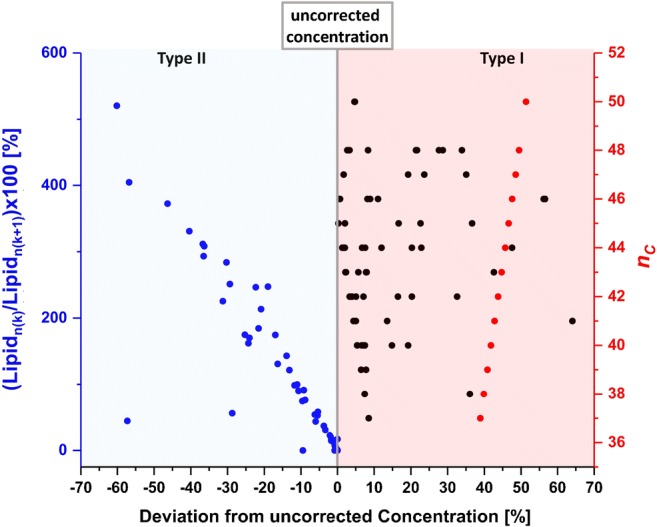
Illustration of deviations in quantified values (plotted as % from uncorrected concentrations, gray line; *x*-axis) arising from isotopic corrections. The effect of lipid total carbon number (*n*_c_; *y*-axis) on type I isotopic correction is shown in red. The influence of the relative concentrations of coeluting lipids that differed by one double bond (shown as the percent of the AUC ratio of the monoisotopic signal of quantified lipid lipid_*n*(*k*)_ and second isotopologue of the lipid with one more double bond lipid_*n*(*k* + 1)_; *y*-axis) on type II correction is shown in blue. The cumulative effect of type I + II corrections is represented by black traces

Type I isotopic correction can be performed by extrapolating the peak area intensities for the corresponding isotopologues based on the intensity of the monoisotopic mass, the natural abundance of ^13^C, and the number of carbons in the molecule [15]. Thus, the magnitude of the correction factor will depend on the number of carbon atoms and will increase with the size of the lipid (Fig. 1, red trace). For instance, the type I isotopic correction factor for PC lipids results in differences relative to uncorrected values ranging from 39.9% for PC(30:0) with 38 C atoms to 51.3% for PC(42:6) with 50 C atoms (ESM Table S2).

#### (3). Type II isotopic correction

Furthermore, accurate lipid quantification is also affected by the overlap of the monoisotopic signal of the quantified lipid and the second isotopologue (*[M + 2]*) of a lipid with one more double bond. Those signals cannot be distinguished at a resolution of 140,000 at *m*/*z* 200 or lower. Therefore, type II isotopic correction has to be applied. First, the peak area of the monoisotopic signal of the lipid of interest (AUC_*n*(*k*)_) and the lipid with one more double bond (AUC_*n*(*k* + 1)_) are determined. Next, the AUC for the second isotopologue (*[M + 2]*) of the lipid with one more double bond is calculated and subtracted from AUC_*n*(*k*)_. The type II isotopic correction is only necessary when lipid *n*(*k*) and lipid *n*(*k* + 1) cannot be chromatographically resolved which is usually the case for HILIC separations but not RPLC. Moreover, the type II correction factor will be strongly influenced by the relative concentrations of coeluting lipids that differed by one double bond (Fig. 1, blue trace), resulting in the higher percent of the difference relative to the uncorrected values for lipid pairs in which the signal intensity of lipid *n*(*k* + 1) is much higher than that of lipid *n*(*k*) (Fig. 1, blue trace, ESM Table S2). Type II corrected values are further subjected to type I isotopic correction discussed above. Therefore, AUC values for monoisotopic signal acquired in HILIC mode were corrected with type II and type I isotopic corrections whereas those acquired in RPLC mode were corrected only with type I (Fig. 1, black trace).

#### (4). Correction for all-ion abundance of deuterated standards

The SPLASH® LIPIDOMIX® contains a non-naturally occurring, deuterated representative for each main lipid class in blood plasma and can therefore be used for quantification of those classes. Determination of the total ion abundance of those deuterated lipids is complicated by the incomplete deuteration of the ISTD which has to be considered in the data processing workflow. Usually, only the integrated peak area of the monoisotopic mass *[M]* of a certain lipid ion is used because it can be determined with the highest sensitivity and accuracy. Isotopologue intensities are then mathematically extrapolated and summed up to determine total ion abundance (similar to type I isotopic correction described above). However, isotopically labeled molecules can have incomplete isotopic enrichment and purity; therefore, non-negligible ion intensities are found also for *[M−1]* and *[M−2]* depending on the number of introduced deuterium atoms and the compounds’ purity. Each of those isotopologues itself has a carbon-dependent ^13^C abundance profile which makes the calculation of all isotopologues using just the monoisotopic mass rather complicated. Additionally, for those calculations, the isotopic enrichment and purity of each ISTD have to be quantified accurately and can vary in between batches. Due to those disadvantages, the total ion abundance is determined by integrating and summing up peak areas of each isotopologue of the deuterated ISTD (ESM Fig. S1), i.e., *[M−2]*, *[M−2]*, *[M]*, *[M + 1]*, *[M + 2]*, and *[M + 3]*.

Eventually, corrected abundances of the native species are multiplied with the concentration of ISTD and divided by the total ion abundance of the ISTD to get the concentration of the native lipid.

Type I isotopic correction depends only on the total carbon number *n*_C_ and, if not performed, can introduce a quantification error of 26% in the case of lipids with *n*_C_ = 20 up to 51% for *n*_C_ = 50. Type II isotopic correction depends on the presence of a lipid signal with one more double bond relative the quantified lipid as well as the signal intensities for both of those signals. For instance, in the case of the 88 PC lipids quantified here with the HILIC MS method, 64 required type II correction. The impact of lipid relative concentrations on type II correction can be exemplified on the PC(38:x) series. For instance, PC(38:6) (76.2 nmol/mL) was corrected for the second isotopologue of PC(38:7) (62.6 nmol/mL) leading to the decrease of the PC(38:6) concentration by only ≈ 10% since both of the lipids were present at similar concentrations. Whereas type II correction for PC(38:3) (15.4 nmol/mL) using the isotopologue of PC(38:4) (99.6 nmol/mL) changed the obtained concentration value by ≈ 57%. Thus, we would like to highlight one more time the importance of correction factors to ensure high accuracy of the quantification results.

### Comparison of lipid quantification using RPLC and HILIC MS

HILIC and RPLC are complementary separation methods [17]. HILIC is separating lipids based on their headgroup polarity leading to coelution of all lipid molecular species of a corresponding subclass and its ISTD. RPLC is separating lipids mainly based on hydrophobicity of their fatty acid chains leading to separation of lipid molecular species over a relatively broad retention time range. Compared to HILIC-based separations, RPLC is capable of separating isomeric lipids within one class. However, here, in order to allow for direct comparison of both methods, the quantities of isomeric lipids from RPLC were summed up.

Only lipids that show good chromatographic retention for both methods were compared. HILIC is well suited for the separation of polar lipids whereas for unpolar lipids such as glycerolipids (GL), ceramides (Cer), and cholesteryl esters (CE), no retention takes place and those lipids elute in the void volume. Furthermore, acidic lipid classes such as phosphatidylserines (PS), phosphatidic acids (PA), and phosphatidylinositols (PI) show a bad retention behavior in HILIC separations due to the presence of several different ionization states in solution [23]. RPLC is well suited for the separation of GL, phospholipids (PL), sphingolipids (SL), and CEs. Therefore, in this study, we compared the capability of HILIC and RPLC to quantify five lipid classes including lysophosphatidylcholines (LPC; 23 species), lysophosphatidylethanolamines (LPE; 7 species), phosphatidylcholines (PC; 88 species), phosphatidylethanolamines (PE; 37 species), and sphingomyelins (SM; 36 species) as the most abundant lipids well separated both by RPLC and HILIC MS methods used in our study (ESM Table S1).

**Fig. 2:**
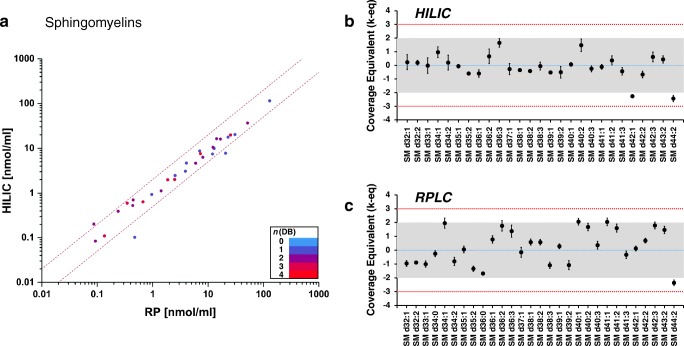
Comparison of sphingomyelin (SM) concentrations in NIST® SRM® 1950 human blood plasma determined using HILIC and RPLC MS workflows (**a**), and comparison of HILIC MS (**b**) and RPLC MS (**c**) results to previously defined consensus values using LipidQC software tool [21]. LipidQC illustrates comparison of normalized lipid quantities to the consensus values. Lipid quantities in accordance with the consensus values (within 95% uncertainty range) lay within gray area of the plot

Out of 204 lipids from five lipid classes considered in this study (LPC, LPE, PC, PE, and SM) with established consensus values, we were able to quantify 184 and 171 lipids by HILIC and RPLC MS workflows, respectively. Lipid concentrations determined using all correction factors mentioned above were compared to the reported consensus values using LipidQC tool [21]. For 36 SM lipids, a good correlation between concentrations determined by HILIC and RPLC MS workflows was observed for nearly all molecular species independent of molecular mass, fatty acid composition, and concentration (Fig. 2). The quantities obtained by both methods are largely consistent with each other with only two lipid molecular species that vary by more than 50% in between both methods. Moreover, SM concentrations measured both by HILIC and RPLC MS were consistent with the consensus values for nearly all molecular species. A similar trend was observed for 7 LPE species quantified in the study (ESM Fig. S2).

**Fig. 3:**
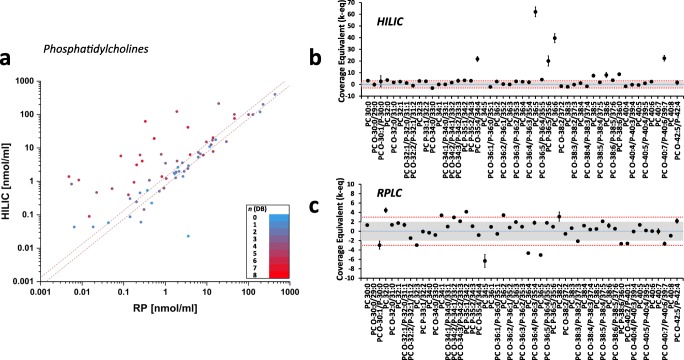
Comparison of phosphatidylcholine (PC) concentrations in NIST® SRM® 1950 human blood plasma determined using HILIC and RPLC MS workflows (**a**) and comparison of HILIC MS (**b**) and RPLC MS (**c**) results to previously defined consensus values using LipidQC software tool [21]. LipidQC illustrates comparison of normalized lipid quantities to the consensus values. Lipid quantities in accordance with the consensus values (within 95% uncertainty range) lay within the gray area of the plot

Concentrations obtained by RPLC MS for 88 PC and 23 LPC lipids were in a good agreement with the consensus values for the majority of lipid species (Fig. 3 and ESM Fig. S3). However, quantities determined by HILIC MS showed that some lipid species were vastly overestimated in comparison to the consensus values (marked by *k-eq* ≥ 10). By plotting lipid quantities determined by HILIC vs RPLC MS, one can see that almost all “overestimated” lipids were characterized by a high unsaturation degree (*n*(*DB*) ≥ 4). A similar trend but to a smaller extent was observed for PE lipids as well (ESM Fig. S4).

### Effect of retention time distribution between lipid and corresponding standard on the quantification results

**Fig. 4:**
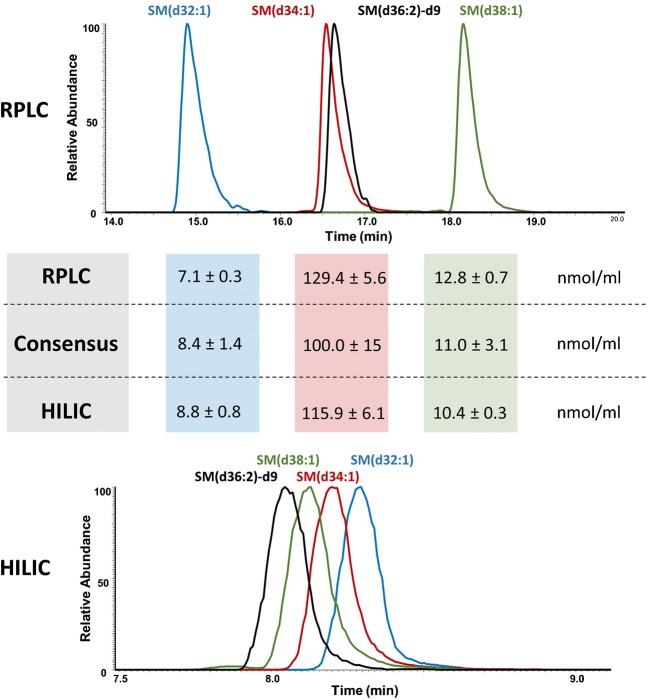
Chromatographic retention behavior and concentrations determined by HILIC and RPLC MS for SM(d32:1), SM(d34:1), SM(d38:1), and ISTD SM(d36:2)-d9 of NIST® SRM® 1950 human blood plasma

Quantification by HILIC MS-derived methods has been stated to provide superior results relative to RPLC MS due to close elution of ISTDs and corresponding lipid molecular species, thus diminishing differential matrix effects [12], whereas in RPLC, lipids of the same class are separated over a broad retention time range exposing ISTD and lipid molecular species to different matrix effects. We therefore addressed the influence of retention time differences between the ISTD and the lipids to be quantified in HILIC and RPLC MS. Three SM species were chosen to display the effect of earlier, later, or coelution with the ISTD in RPLC (Fig. 4).

Separation by HILIC and RPLC is exemplified for the lipids SM(d32:1), SM(d34:1), SM(d38:1), and the corresponding ISTD SM(d36:2)-d9. In RPLC, SM(d32:1) is eluting before (Δ_tR_ = 1.8 min) and SM(d38:1) is eluting after (Δ_tR_ = 1.6 min) ISTD SM(d36:2)-d9 whereas in HILIC, close elution with a maximum retention time difference of 0.3 min to the ISTD can be observed. Quantification of those signals with all applied correction factors yields similar concentrations between HILIC and RPLC well suited within the 95% confidence interval calculated by LipidQC. Furthermore, those results were compared to SM(d34:1) that is coeluting with the ISTD in both applied separation modes. Slightly higher concentrations are determined in the case of RPLC but as shown previously (Figs. 2 and 3) remains within the 95% confidence interval. In conclusion, coelution of ISTD and lipids is not obligatory to obtain accurate quantification for lipid species addressed in this study.

### Effect of lipid concentrations on the HILIC MS quantification results

Previous direct injection experiments on equimolar mixtures of lipids with different degrees of unsaturation and fatty acyl chain lengths demonstrated that lipids with a higher number of double bonds as well as lipids with decreasing chain length show higher intensities [26, 27]. This effect was dependent on the total lipid concentration during ionization and could be diminished by diluting lipid mixtures prior to injection. The difference in the surface activity during ionization was proposed to be the main factor explaining higher signal intensities for unsaturated lipids. Additionally, it was proposed that double bonds in lipids could weaken the intermolecular interactions on the droplet surface [26]. Since all lipid species for a certain lipid class are coeluting in HILIC, it might be compared to direct injection experiments in which no separation prior to MS analysis is performed. Moreover, PC lipids for which “overestimated” concentrations were measured have the highest total plasma concentrations followed by SM, LPC, PE, and LPE lipid classes. For instance, total SM concentration in plasma is around three times lower than that of PC. Furthermore, all measured SM lipids had *n*(*DB*) ≤ 3, whereas PC lipid species contained up to eight double bonds.

Another point to be considered is the contribution of added ISTD lipids to electrospray saturation effects. However, here, the concentration of PC ISTD in plasma was ≈ 19 μM (corresponding to 3.2 pmol on the column) and thus was comparable to the medium abundant PC lipids. Thus, it is possible that electrospray saturation and its effect on MS response are unlikely to be attributed to PC ISTD when compared to abundant PC species (e.g., PC 34:2 with 240 μM/67.7-pmol on the column or PC 36:4 with 150 μM/27.3 pmol on the column) with potentially higher ESI-MS response due to increased surface activity determined by a higher number of double bonds [28–30].

Here, to test the effect of total PC concentration on the “overestimation” of unsaturated molecular species, lipid extract was quantified by HILIC MS using several consecutive dilutions with the total amounts of lipid extracts equivalent to 0.15, 0.1, 0.05, 0.01, and 0.005 μL of original human blood plasma.

**Fig. 5:**
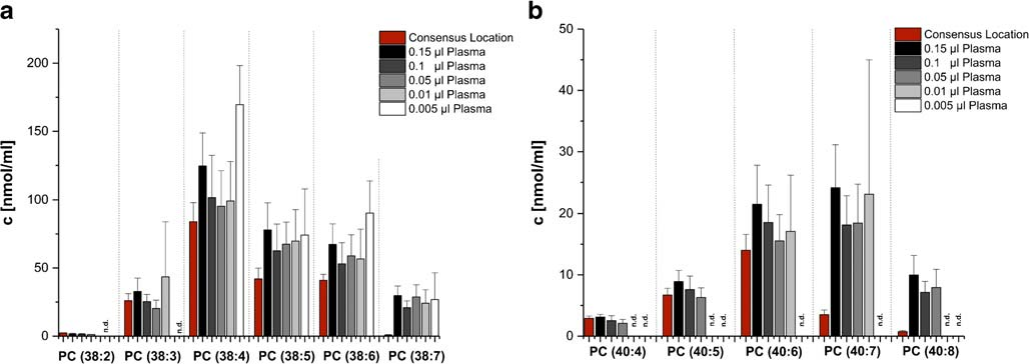
Comparison of lipid concentrations determined by HILIC MS for **a** PC(38:2) to PC(38:7) and **b** PC(40:4) to PC(40:8) consensus values reported to these species (red bars) using different loading amounts of lipid extract equivalent to 0.15, 0.1, 0.05, 0.01, and 0.005 μL of original NIST® SRM® 1950 human blood plasma

Effect of different loading amounts on lipid quantification by HILIC MS exemplified here for two PC series (Fig. 5). For PC lipids with 38 carbon atoms, good fit to the consensus values as well as RPLC MS quantitative results was observed for species with *n*_DB_ ≤ 4 independent of the dilution factor. However, concentrations of PC(38:5) until PC(38:7) were “overestimated” using the highest loading amount (0.15 μL of blood plasma). This effect was slightly reduced for PC(38:5) and PC(38:6), but was still evident for PC(38:7) (Fig. 5a). A similar trend can be observed for the series PC(40:x) where the effect of “overestimation” was reduced by using diluted lipid extract for PC(40:6) but remained unchanged for PC(40:7) and PC(40:8) (Fig. 5b). Furthermore, decreasing sample loading led to higher standard deviations due to the integration of signals of very low intensity.

### Effect of different ratios of quantified lipids and internal standard

Diluting lipid extracts before HILIC analysis did not significantly improve the accuracy of quantification for highly unsaturated PC. We then evaluated if the difference in intensity between the quantified lipid and corresponding ISTD could explain the observed “overestimation.” Previously, using the direct injection approach, it was proposed that a certain lipid can be quantified only if its intensity lies within the range of 20–500% of the intensity of the corresponding ISTD signal [15].

**Fig. 6:**
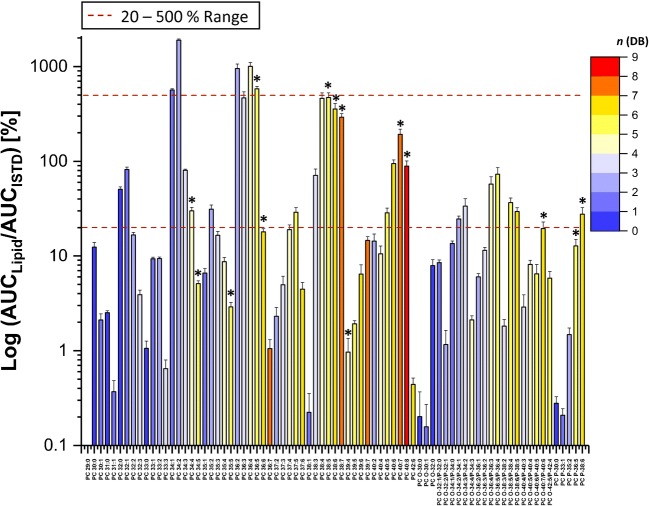
AUC ratio for quantified phosphatidylcholines (PC) and the corresponding ISTD measured by HILIC MS. The range of the ratio within 20 to 500%, required for the accurate quantification as reported in the previous study [15], is marked with the red dotted line. PC unsaturation degree is presented as a color range from blue (zero double bonds; *n*(DB) = 0) to red (nine double bonds; *n*(DB) = 9). Values with *k-eq* ≥ 6 are marked with *

Using corresponding deuterated PC lipid present in SPLASH® LIPIDOMIX®, we determined those ratios for all PC lipids. Intensities of blood plasma PC lipids were within the range from 1 to 600% of the ISTD (Fig. 6). However, no correlation between “overestimation” and intensity values relative to ISTD was observed. In fact, majority of highly unsaturated PC lipids for which substantial differences between HILIC and RPLC MS methods were measured were within the range of intensity ratios suitable for quantification according to the previous report [15].

It was previously reported that isobaric interference of the [M + Na]^+^ signal of a lipid with 3 less double bonds and 2 less carbons with the [M + H]^+^ adduct of the quantified lipid may lead to overestimation in quantitative results [31–33]. For example, the [M + H]^+^ adduct of PC 40:7 (*m*/*z* 832.5851) overlaps with the [M + Na]^+^ adduct of PC 38:4 (*m*/*z* 832.5827). Those signals to be accurately differentiated would need a mass accuracy below 3 ppm and a resolving power of ≈ 350,000 that even modern high-resolution accurate mass MS cannot routinely deliver. Those artifacts occur only if lipids with *n*_DB_ ≥ 3 are present in the studied biological matrix which was the case for PC, LPC, and PE but not SM and LPE lipids in human plasma studied here and therefore could potentially explain the observed “overestimation” effect.

Assessing those isobaric interferences by HILIC MS was not possible at the analytical conditions used here (mass accuracy of 5 ppm and MS resolving power of 73,300 at *m*/*z* 832.5827), so we estimated the contribution of isobaric overlap by calculating the sum of [M + Na]^+^ and [M + H]^+^ of isobarically overlapping lipid signals in RPLC MS (ESM Table S3). For instance, concentrations determined for PC 40:7 [M + H]^+^ by RPLC MS and HILIC MS corresponded to 3.49 μM and 43.97 μM, respectively. To mimic the HILIC MS situation where signals of PC 40:7 [M + H]^+^ and PC 38:4 [M + Na]^+^ are indistinguishable and thus quantified together, those two signals, well separated by RPLC, were quantified and summed, providing the value of 17.59 μM, which could explain only 35% of “overestimated” values obtained by HILIC MS. Overall, those values varied between 5 and 94% for 14 analyzed PC lipids (ESM Table S3). However, it should be considered that it is rather rough approximation, since different solvent systems, different elution times, and isomer separation in RPLC could lead to a different relative intensity of the [M + Na]^+^ to [M + H]^+^ adducts for different lipid species.

## Conclusion

The lipidomics community nowadays aims to establish guidelines “to develop common standards for minimum acceptable data quality and reporting for lipidomics data” with a focus on providing reliable and accurate quantitative readouts for lipid species in natural lipidomes [34]. To aid the question of selecting the optimal LC–MS method for lipid quantification, we performed the systematic comparison between two HRAM MS-based quantification workflows based on HILIC and RPLC MS by quantifying 191 lipids from five lipid classes (LPC, LPE, PC, PE, and SM) in human blood plasma using deuterated standards in the “one ISTD-per-lipid class” approach. Quantification workflow followed the recommendation of the Lipidomics Standard Initiative (https://lipidomics-standards-initiative.org/guidelines/lipid-species-quantification) by considering two types of isotopic corrections as well as correction for the isotopic purity of deuterated standards used for the quantification. We provide a detailed comparison of quantification results with and without each type of correction and highlight the necessity of utilizing these correction factors to ensure high accuracy of the results.

Lipid concentrations determined using HILIC and RPLC MS were compared to each other as well as to the consensus values established for the same lipid species in NIST SRM 1950 human blood in a previously published multi-laboratory study [2]. It has been stated that different separation modes (HILIC vs RPLC) have varying suitability for the quantification of lipids from biological matrices. HILIC is believed to be better suited for lipid quantification since native lipids coelute with corresponding ISTD, thus reducing matrix effects originating from different solvent compositions during ionization [12, 13]. Following this logic, RPLC is not suited for lipid quantification since ISTD usually elute in a quite different solvent composition and therefore suffer from increasing solvent-dependent matrix effects. We have shown that HILIC and RPLC, despite the obvious difference in matrix effects, yield similar values for PE, LPE, and SM consistent with the reported consensus values. Yet, quantification of (L)PC lipids showed higher quantities in the HILIC method compared to RPLC MS and consensus values, especially in the case of highly unsaturated PC lipids.

Neither reduced lipid loading nor the differences between intensities of the ISTD and quantified lipid signals, or isotopic overlap with [M + Na]^+^ adducts of lipids with 3 less double bonds and 2 less carbons could fully explain observed “overestimation.” As it has been stated before, the differences in lipid MS response are dependent on distinct molecular features such as unsaturation level, acyl/alkyl chain length, and bond types [19, 26, 27, 35–37] and thus can only be properly addressed by application of a response factor approach or the use of several different ISTD per class (i.e., with different chain lengths and number of double bounds) to make up for differential response. However, so far, almost no equimolar mixtures of isotopically labeled molecular species with different degrees of unsaturation and fatty acyl/alkyl chain length for a given specific lipid class are commercially available and thus generation of response factors is tedious. One possible solution is provided by Lipidyzer™ Platform kits including 50 ISTD covering 13 lipid classes.

Furthermore, comparison of the measured concentrations to the reported consensus values might be biased as there is no information available on which separation modes, mass analyzers, or ISTD have been used for establishing them. If it is the case that mostly RPLC MS was used for generation of the consensus values then those quantities have to be reevaluated by incorporating also HILIC-based quantification approaches.

## Electronic supplementary material

ESM 1 (PDF 562 kb)

ESM 2 (XLSX 198 kb)

## Abbreviations

AUC: Area under the curve
CE: Cholesteryl ester
Cer: Ceramides
GL: Glycerolipids
HILIC: Hydrophilic interaction chromatography
HRAM: High-resolution accurate mass
*i*-PrOH: Isopropanol
ISTD: Internal standard
LC-MS: Liquid chromatography–mass spectrometry
LPC: Lysophosphatidylcholine
LPE: Lysophosphatidylethanolamine
*m*/*z*: Mass to charge ratio
MeCN: Acetonitrile
MeOH: Methanol
MS: Mass spectrometry
MS^2^: Tandem mass spectrometry
MTBE: Methyl-*tert*-butyl ether
NH_4_HCO_2_: Ammonium formate
NH_4_OAc: Ammonium acetate
PA: Phosphatidic acid
PC: Phosphatidylcholine
PE: Phosphatidylethanolamine
PI: Phosphatidylinositol
PRM: Parallel reaction monitoring
PS: Phosphatidylserine
RPLC: Reversed-phase liquid chromatography
RSD: Relative standard deviation
SL: Sphingolipids
SM: Sphingomyelin
