# Supplementary material for: Evaluation of lipid quantification accuracy using HILIC and RPLC MS on the example of NIST® SRM® 1950 metabolites in human plasma
Source: Anal Bioanal Chem. 2020 Apr 2;412(15):3573–84. doi: 10.1007/s00216-020-02576-x (PMC7220885; doi:10.1007/s00216-020-02576-x)
Supplement: Supplementary file 1 — (PDF 562 kb) [file 216_2020_2576_MOESM1_ESM.pdf]

Analytical and Bioanalytical Chemistry

Electronic Supplementary Material

Evaluation of lipid quantification accuracy using HILIC and RPLC MS on the example of NIST® SRM® 1950 metabolites in human plasma

Mike Lange, Maria Fedorova

Additional file available under 10.1007/s00216-020-02576-x

Separate Excel file:

Table S1 Lipid concentrations (nmol/mL) and retention times (tR) determined by RPLC and HILIC MS workflows in NIST® SRM® 1950 human blood plasma. Values for five technical replicates, average as well as standard deviation and relative standard deviation are provided for each lipid. All given values were subjected to all correction factors described in the study. For comparison consensus locations and standard uncertainties as previously reported are presented [2]

Table S2 Differences (%) to uncorrected values after application of factors for all-ion abundance of deuterated standards, type I, type II isotopic correction and combination of all corrections for RPLC and HILIC MS quantification workflows. Negative values obtained after corrections are marked in red

Table S3 Obtained concentrations of overlapping [M+H]+ and [M+Na]+ adducts in RPLC separations and comparison to obtained concentrations with HILIC MS. Values added are marked in orange

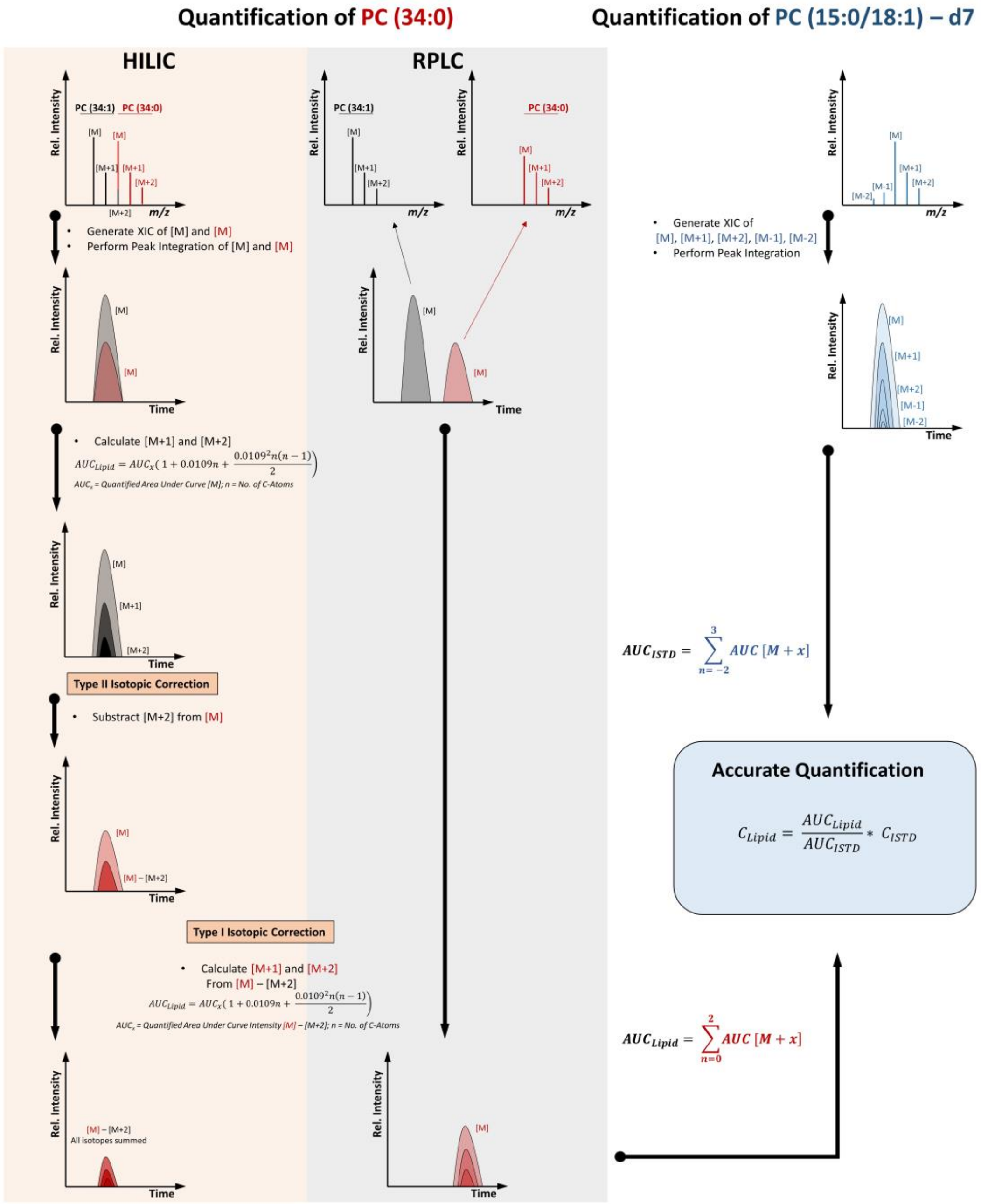

Fig. S1 Detailed graphical representation of lipid quantification strategy considering Type I and Type II isotopic corrections as well as correction for all-ion abundance of deuterated standard in HILIC and RPLC-MS lipidomics analysis

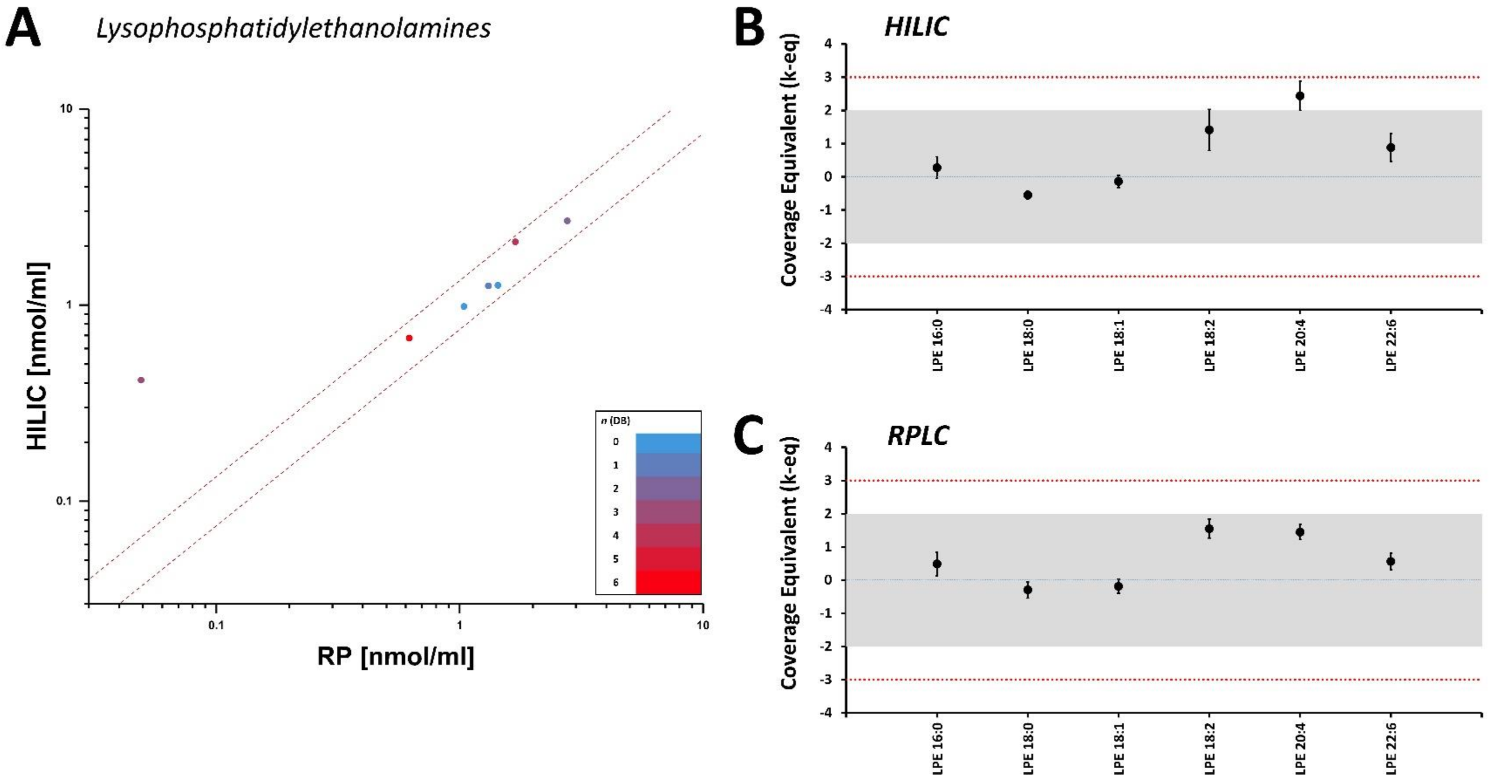

Fig. S2 Comparison of lysophosphatidylethanolamine (LPE) concentrations in NIST® SRM® 1950 human blood plasma determined using HILIC and RPLC MS workflows (A), and comparison of HILIC MS (B) and RPLC (C) results to previously defined consensus values using LipidQC software tool. LipidQC illustrates comparison of normalized lipid quantities to the consensus values. Lipid quantities in accordance to the consensus values (within 95% uncertainty range) lay within gray area of the plot

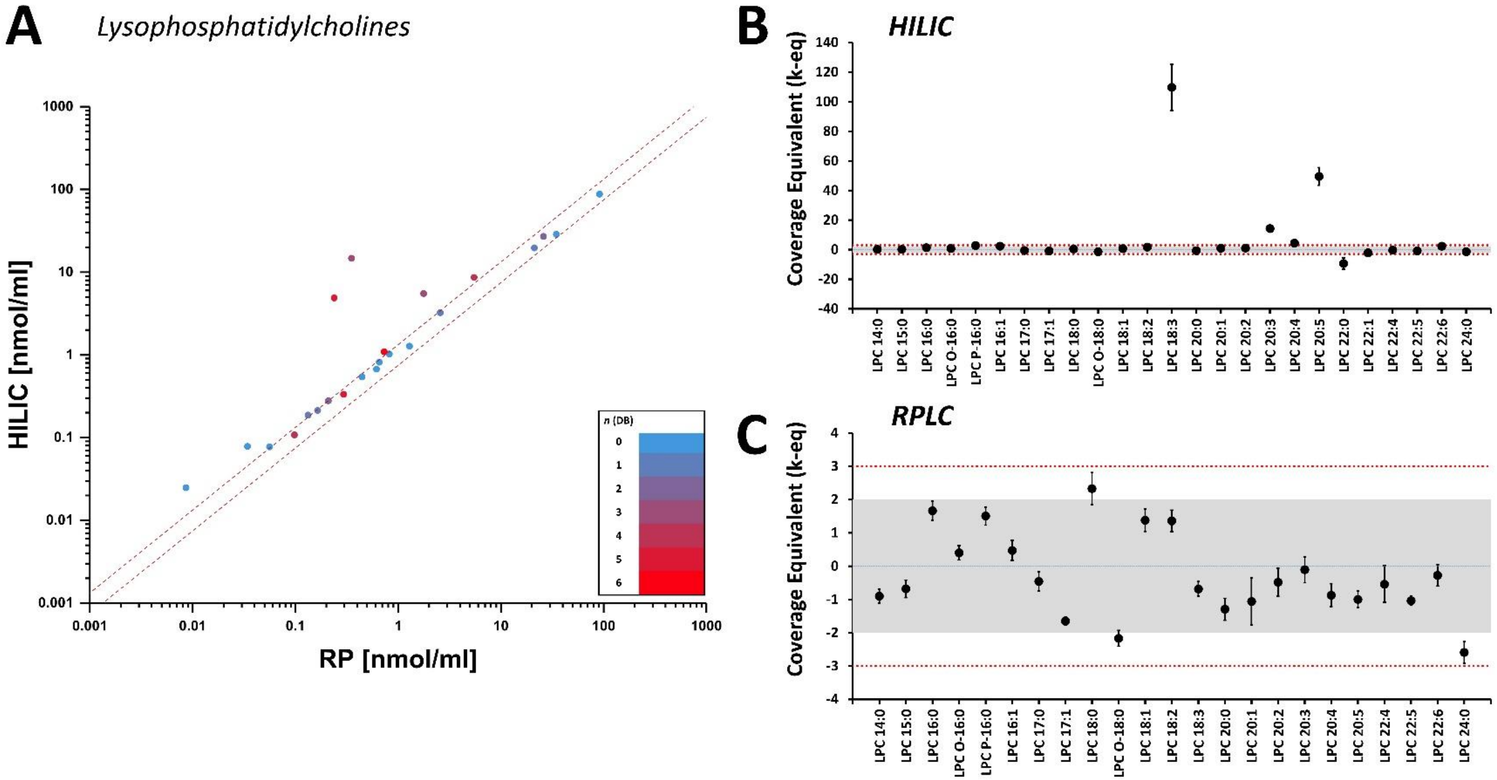

Fig. S3 Comparison of lysophosphatidylcholine (LPC) concentrations in NIST® SRM® 1950 human blood plasma determined using HILIC and RPLC MS workflows (A), and comparison of HILIC MS (B) and RPLC (C) results to previously defined consensus values using LipidQC software tool. LipidQC illustrates comparison of normalized lipid quantities to the consensus values. Lipid quantities in accordance to the consensus values (within 95% uncertainty range) lay within gray area of the plot

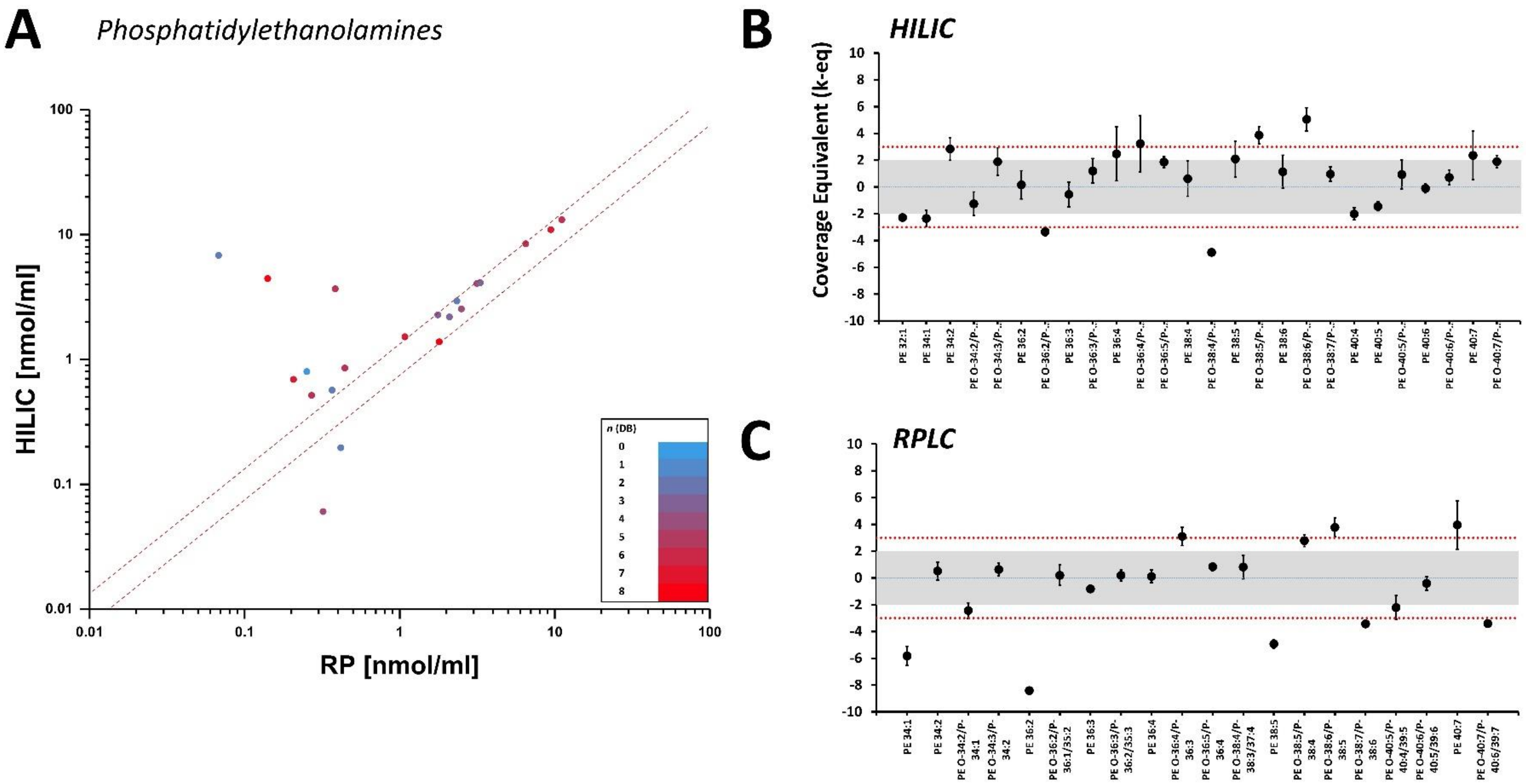

Fig. S4 Comparison of phosphatidylethanolamine (PE) concentrations in NIST® SRM® 1950 human blood plasma determined using HILIC and RPLC MS workflows (A), and comparison of HILIC MS (B) and RPLC MS (C) results to previously defined consensus values using LipidQC software tool. LipidQC illustrates comparison of normalized lipid quantities to the consensus values. Lipid quantities in accordance to the consensus values (within 95% uncertainty range) lay within gray area of the plot
